# Vertical stratification and functional coupling of antibiotic resistance and carbon metabolism in thermokarst lake sediments

**DOI:** 10.1093/ismeco/ycag107

**Published:** 2026-05-08

**Authors:** Ze Ren, Mingfeng Zhao, Ruoting Chen, Xinyun Zhang

**Affiliations:** Key Laboratory of Lake and Watershed Science for Water Security, Nanjing Institute of Geography and Limnology, Chinese Academy of Sciences, Nanjing 210008, China; College of Resources and Environment, University of Chinese Academy of Sciences, Beijing 100049, China; Key Laboratory of Lake and Watershed Science for Water Security, Nanjing Institute of Geography and Limnology, Chinese Academy of Sciences, Nanjing 210008, China; College of Resources and Environment, University of Chinese Academy of Sciences, Beijing 100049, China; School of Environment, Nanjing Normal University, Nanjing 210023, China; Key Laboratory of Lake and Watershed Science for Water Security, Nanjing Institute of Geography and Limnology, Chinese Academy of Sciences, Nanjing 210008, China; College of Resources and Environment, University of Chinese Academy of Sciences, Beijing 100049, China; College of Hydrology and Water Resources, Hohai University, Nanjing 210098, China; Key Laboratory of Lake and Watershed Science for Water Security, Nanjing Institute of Geography and Limnology, Chinese Academy of Sciences, Nanjing 210008, China; College of Resources and Environment, University of Chinese Academy of Sciences, Beijing 100049, China

**Keywords:** antibiotic resistance genes, sediment cores, carbohydrate metabolism, vertical distribution, thermokarst lakes

## Abstract

Thermokarst lakes are biogeochemical hotspots and reservoirs of antibiotic resistance genes (ARGs), yet their vertical organization remains poorly understood. Here, we investigated the vertical stratification of ARGs in sediment cores from thermokarst lakes on the Qinghai–Xizang Plateau, quantifying their distribution and associations with mobile genetic elements (MGEs) and carbohydrate-active enzymes (CAZymes). The results revealed pronounced vertical differentiation, with ARG richness decreasing but β-diversity increasing with depth. A total of 386 ARGs were identified, of which 39% increased and 22% decreased significantly along the depth gradient. Multidrug and glycopeptide resistance genes dominated the profiles, while macrolide, tetracycline, and fluoroquinolone resistance were most abundant overall. MGEs, primarily transposase and recombinase genes, were strongly correlated with ARGs, underscoring horizontal gene transfer as a key mechanism for their persistence and dispersal. Co-occurrence analyses further revealed both positive and negative associations between ARGs and CAZymes, indicating synergistic and antagonistic couplings between antibiotic resistance and microbial carbon metabolism. Genes involved in energy-efficient carbon degradation (e.g. glycoside hydrolases and glycosyltransferases) were positively correlated with resistance genes enhancing stress tolerance, whereas negative interactions reflected trade-offs between carbon utilization and resistance maintenance. These findings demonstrate that ARGs are vertically structured and functionally integrated within microbial metabolic networks, providing new insights into their ecological roles in thermokarst lakes.

## Introduction

In the Anthropocene, antibiotic resistance genes (ARGs) have emerged as a global environmental and public health concern [1], primarily due to their ability to spread through horizontal gene transfer (HGT) and confer resistance to a wide range of antibiotics [2, 3]. However, ARGs have naturally evolved over millions of years as microbial defense mechanisms [4, 5], widely distributed across various natural ecosystems, even in cryosphere environments such as glaciers [6, 7], permafrost [5, 8], and thermokarst lakes [9]. Thermokarst lakes, formed by the thaw and collapse of ice-rich permafrost, are dynamic freshwater systems distributed across the Arctic, sub-Arctic, and the Qinghai–Xizang Plateau (QTP) [10, 11]. These lakes are expanding rapidly under climate warming, reshaping hydrological connectivity and biogeochemical cycling [12], as well as acting as ARG’s hotspot [9, 13]. The deep sediments of thermokarst lakes remain largely free from direct anthropogenic pollution, providing a natural archive to reconstruct the baseline occurrence and evolutionary trajectories of ARGs in pristine environments [14].

Thermokarst lake sediments cores record long-term environmental and microbial changes [14, 15], offering an ideal framework to trace vertical variations in ARG abundance and diversity. Sediment depth gradients are widely recognized to be associated with shifts in redox state, organic matter quality, and nutrient availability, which in turn influence microbial community composition and functional potential [16, 17]. Vertical stratification reflects the combined influence of historical deposition and ongoing ecological adaptation, where deeper layers preserve legacy microbial and ARG signatures while also undergoing continuous selection under prevailing redox and energy constraints. Mobile genetic elements (MGEs), including transposases, integrons, and conjugative transfer proteins, further facilitate the horizontal transfer of ARGs [18, 19]. These MGEs can mediate the exchange of ARGs between diverse microbial taxa, enhancing the potential for multidrug resistance [20]. Understanding the co-distribution of ARGs and MGEs along sediment profiles is thus essential for identifying the environmental conditions that promote ARG persistence and mobility.

Beyond antibiotic resistance, ARGs may exert broader ecological functions by interacting with microbial metabolic pathways, particularly those linked to organic carbon cycling [21]. Permafrost landscapes store nearly half of Earth’s soil organic carbon and are particularly susceptible to climate change [22, 23]. Thermokarst lakes act as biogeochemical hotspots within these landscapes, hosting diverse microbial consortia that drive carbon transformation and greenhouse-gas production [24, 25]. Their sediments contain a mixture of allochthonous and autochthonous organic compounds derived from plants, algae, and microbes [26, 27]. The degradation of these complex substrates relies on carbohydrate-active enzymes (CAZymes) that catalyze polysaccharide depolymerization [28, 29]. However, the potential coupling between ARGs and CAZymes, representing microbial resistance and metabolic capabilities, remains largely unexplored. Such linkages could reveal functional co-selection processes that integrate antibiotic resistance with carbon metabolism in cryosphere ecosystems.

The rapid thawing of permafrost under climate change intensifies the relevance of these interactions. Permafrost degradation releases vast pools of organic carbon, nutrients, and potentially ancient ARGs, reactivating dormant microbial communities and altering modern gene exchange networks [30, 31]. On the QTP, the world’s largest high-altitude permafrost region, approximately 19.0 ± 6.6 Pg of soil organic carbon is stored in permafrost [32], which is undergoing rapid thaw, driving the widespread formation and expansion of thermokarst lakes [33]. These lakes provide an exceptional natural laboratory to investigate the evolution, mobilization, and ecological roles of ARGs. Here, we integrate metagenomic and functional analyses of sediment cores from QTP thermokarst lakes to (i) quantify vertical patterns of ARG abundance and diversity, (ii) examine associations between ARGs and MGEs, and (iii) explore potential co-selection between ARGs and CAZymes. By integrating these dimensions, we reveal how microbial resistance, mobility, and metabolic potential co-evolve in thermokarst lake sediments, offering new insights into the ecological and biogeochemical roles of ARGs in a warming cryosphere.

## Materials and methods

### Study area and field sampling

The QTP, referring to as the Earth’s third pole, is characterized by permafrost covering roughly 40% of its land surface (Fig. S1) [34]. Projections suggest that by 2100, 40%–78% of the QTP’s permafrost may disappear [33]. Currently, the QTP permafrost regions contain approximately 161 300 thermokarst lakes (ranging from 500 m^2^ to 3 km^2^ in size) with a combined area of 2825 km^2^ [11].

This study was conducted on the QTP in July 2023, focusing on 19 thermokarst lakes located in the Yellow River Source Area (Fig. S1). In thermokarst lakes, bottom sediments may comprise both recently deposited materials and thawed permafrost substrates exposed during lake formation; therefore, we use the term “sediments” here to broadly describe these deposits [12]. Sediment cores were extracted from each lake, with core lengths varying between 30 cm and 60 cm, depending on the sampling conditions and equipment capabilities. Each core was sectioned into 1 cm intervals to ensure precise depth-resolved analysis of microbial and sediment properties. Although cores were sectioned at 1 cm intervals, metagenomic analyses were performed on selected depths, with finer resolution in surface sediments (0–20 cm at 5 cm intervals) and broader spacing in deeper layers (>20 cm at 10 cm intervals), to capture major environmental gradients while ensuring analytical feasibility. Microbial samples were taken from specific depths: 0–1 cm (d01), 4–5 cm (d05), 9–10 cm (d10), 14–15 cm (d15), 19–20 cm (d20), 29–30 cm (d30), 39–40 cm (d40), 49–50 cm (d50), and 59–60 cm (d60), allowing for a comprehensive examination of microbial community composition across various sediment layers. In total, 144 sediment samples were collected from all the lakes, providing robust coverage of vertical microbial stratification and environmental gradients within the sediments. Microbial samples were immediately transferred into 5-ml sterile centrifuge tubes, which were stored on dry ice during fieldwork. The remaining sediment portions were frozen on-site and later freeze-dried in the laboratory for further examination.

### DNA extraction, sequencing, and data processing

Genomic DNA was extracted from the sediment samples using DNA extraction kits provided by Guangdong Magigene Biotechnology Co., Ltd. (Guangzhou, China). The quality and integrity of the extracted DNA were verified by 1% agarose gel electrophoresis, and the concentration and purity were measured using a Qubit 4.0 fluorometer and a NanoDrop One spectrophotometer (Thermo Fisher Scientific, Waltham, USA). Only DNA samples that passed quality control checks were processed for sequencing using the ALFA-SEQ DNA Library Preparation Kit (Guangdong Magigene Biotechnology Co., Ltd.). The prepared libraries were sequenced on the Illumina NovaSeq 6000 platform using PE150 sequencing.

The raw sequencing reads were processed with Trimmomatic (v0.39) [35] to remove low-quality reads, generating clean data suitable for downstream analyses. MEGAHIT (v1.2.9) [36] was used for assembling the clean data into scaffolds, which were further fragmented at N-connection sites, producing N-free Scaftigs. MEGAHIT was then used again to compare each Scaftig with the clean data from all individual samples, identifying paired-end reads not incorporated in the initial assembly. These unutilized reads from all samples were pooled together for a mixed assembly, using the same parameters as the single-sample assemblies. The mixed scaffolds were also fragmented at N-connection sites, producing a set of N-free Scaftigs, with those longer than 500 bp selected for statistical analysis.

Open reading frame (ORF) prediction was conducted on Scaftigs (≥500 bp) from both individual and mixed assemblies using Prodigal (v2.6.3) [37]. To reduce redundancy, MMseqs2 was employed, resulting in a non-redundant initial gene catalog, with genes defined as distinct, continuous nucleotide sequences. Clustering of genes was performed at 95% sequence identity and 90% coverage, with the longest sequence from each cluster selected as the representative. The clean data from each sample were then mapped to the gene catalog using BBMap [38], generating read counts for each gene in each sample. Gene abundance in each sample was determined based on these mapped read counts and gene lengths.

### Annotation of taxonomy, antibiotic resistance genes, mobile genetic elements, and carbohydrate-active enzymes

Taxonomic classification of the microbial communities was performed by aligning the predicted ORFs from each sample against the NCBI NR database using Diamond BLASTX. When multiple alignment results were available for a single sequence, the lowest common ancestor (LCA) algorithm in MEGAN software was applied for species-level annotation, with an E-value threshold of 1e-10. From the LCA results and the gene abundance or gene average depth tables, taxonomic abundance tables were generated for each taxonomic level, from kingdom to species. To identify ARGs within the microbial communities, the ORFs were aligned against the Comprehensive Antibiotic Resistance Database (CARD) [39] using the Diamond BLASTX tool with an E-value cutoff of 1e-5. ARG abundance in each sample was determined by summing the read counts mapped to the corresponding ARGs, normalized by gene length. To assess the potential for HGT, the ORFs were searched against the MGEs database (MGEs90) using Diamond BLASTX with an E-value threshold of 1e-5. To examine the metabolic potential related to organic matter degradation, CAZymes were annotated against the CAZymes databases [29].

### Statistical analyses

All statistical analyses were conducted in R 4.3.1 using the packages ‘vegan’, ‘phyloseq’, ‘betapart’, ‘lme4’, ‘nlme’, ‘mgcv’, ‘Hmisc’, ‘igraph’, and ‘qgraph’. To evaluate the vertical trends of ARGs, antibiotic types, and CAZymes across sediment depths, Spearman correlation analysis was performed. False discovery rate (FDR) adjustments were applied using the Benjamini–Hochberg (BH) method, with a significance threshold of FDR < 0.05. Heatmaps were constructed to visualize the distribution patterns of ARGs, antibiotic types, and CAZymes at different sediment depths, providing a clear depiction of their stratification along the vertical profile. Alpha diversity (richness) was calculated for each 1-cm sediment interval, and depth-dependent trends were assessed using linear mixed-effects models (LMMs) with lake identity as a fixed effect. Beta diversity (Bray–Curtis dissimilarities) was used to quantify compositional turnover across depths and lakes, with analysis of similarities (ANOSIM) tests evaluating significance. The vertical trends of beta diversity (among lakes with same depth and among depths within a lake) were also assessed using LMMs with lake identity as a fixed effect. Co-occurrence relationships between ARGs and MGEs, as well as between ARGs and CAZymes, were evaluated using Spearman’s correlation. Correlation *P*-values were adjusted using the BH method for FDR control. Only strong correlations (Spearman’s correlation coefficient |*R*| > 0.5) and significant associations (FDR < 0.05) were considered to construct the co-occurrence networks. The networks were visualized using Gephi 0.10 software.

## Results and discussion

### Vertical distribution and alpha diversity of antibiotic resistance genes in sediment profiles

**Figure 1:**
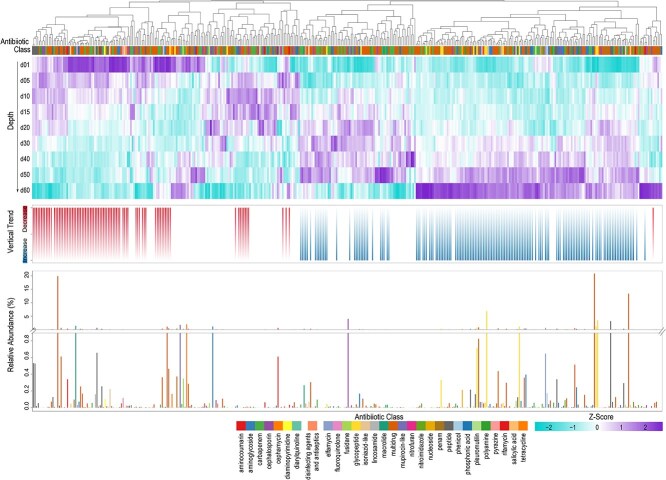
Vertical distribution, relative abundance, and changing trends of ARGs across sediment depth profiles.

**Figure 2:**
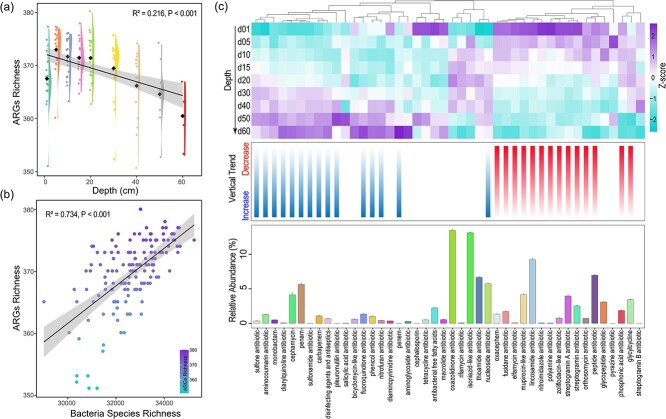
(a) ARGs alpha diversity changing trend along sediment depth. (b) Relationship between ARG richness and bacterial species richness. (c) Vertical distribution, relative abundance, and changing trends of ARGs types.

Across the 19 thermokarst lakes, we identified 386 distinct ARGs spanning 44 resistance classes from all the samples across different lakes and sediment depths. The dominant ARGs across all lakes and depths were *abcA, Abau_AbuO, aadT*, and *van*, each contributing >5% of total relative abundance (Fig. 1). Approximately 80% of ARGs were detected across all samples, with 151 ARGs (39%) increasing and 85 ARGs (22%) decreasing in relative abundance with depth (Fig. 1), highlighting pronounced vertical differentiation. In addition, ARG richness declined significantly with sediment depth (*R*^2^ = 0.41, *P* < .001, Fig. 2A).

This stratification reflects strong environmental filtering along physicochemical gradients, a canonical ecological process governing community assembly in stratified environments [16, 40]. Surface layers likely integrate multiple ARG sources, including microbial inputs from thaw water, wildlife, or atmospheric deposition, leading to higher richness and stochastic assemblages [6, 41]. In contrast, deep anoxic strata harbor stable, low-diversity resistomes dominated by intrinsic or ancient genes, consistent with niche differentiation under persistent redox stratification [16, 40]. Positive correlations between ARG richness and bacterial α-diversity (Fig. 2B) further suggest that resistome composition is tightly coupled to microbial community structure, reflecting environmentally structured assembly processes observed in soils and sediments [42]. The persistence of diverse ARGs in minimally disturbed sediments implies that antibiotic resistance is an ancient and ecologically embedded trait, not solely a product of human activity. Ancient permafrost and cryosols contain ARGs dating back >30 000 years [5, 43], supporting the notion of a “natural resistome” shaped by microbial competition and the endogenous production of secondary metabolites [44, 45]. The widespread occurrence of multidrug and glycopeptide resistance across all depths (Fig. 1) further indicates that these traits are maintained through evolutionary advantages unrelated to synthetic antibiotic exposure, such as defense against naturally produced antimicrobials or adaptation to nutrient limitation, consistent with selection under resource limitation and microbial competition [46]. Although environmental parameters such as redox potential, nutrient availability, and organic matter composition were not directly measured in this study, such gradients are well established in stratified sediments and are widely recognized as fundamental drivers of microbial community assembly [16, 40]. ARGs conferring resistance to macrolides, tetracyclines, fluoroquinolones, and β-lactams were the most abundant (Fig. 2C). Approximately 34% of resistance classes increased with depth, whereas 32% decreased, reinforcing a balance between legacy ARG accumulation and selective retention of metabolically relevant genes. Microbial taxa in anoxic deeper layers may naturally synthesize macrolides or glycopeptides to regulate interspecies interactions [47, 48]. Thus, increasing ARG abundance with depth likely reflects endogenous selection by naturally produced antibiotics and redox-driven metabolic competition, not anthropogenic contamination. Consistent with this interpretation, multidrug-resistance genes accounted for >30% of total ARGs, representing the dominant functional category across all sites (Fig. S2). Such pervasive multidrug profiles suggest broad-spectrum resistance as a survival strategy in energy-limited, fluctuating environments, where maintaining versatile defense systems outweighs the metabolic cost, reflecting metabolic trade-offs in resource allocation [49, 50]. The ubiquity of these genes across depths also points to HGT as a critical mechanism sustaining functional redundancy within the resistome [51, 52].

Mechanistically, antibiotic inactivation and target-alteration pathways dominated the vertical profiles (Fig. S2). Genes encoding β-lactamases and aminoglycoside-modifying enzymes decreased with depth, whereas those mediating target modification (e.g. *rpoB, van operons*) increased. This trend suggests a transition from reactive detoxification near the surface to structural and regulatory adaptations in deeper sediments, potentially reflecting the shift from dynamic to energy-conserving microbial lifestyles [53, 54]. Such mechanistic shifts mirror an ecological succession in resistance strategies, where metabolic specialization under persistent anoxia favors intrinsic, evolutionarily stable forms of resistance.

### Spatial heterogeneity and compositional turnover of antibiotic resistance gene profiles

**Figure 3:**
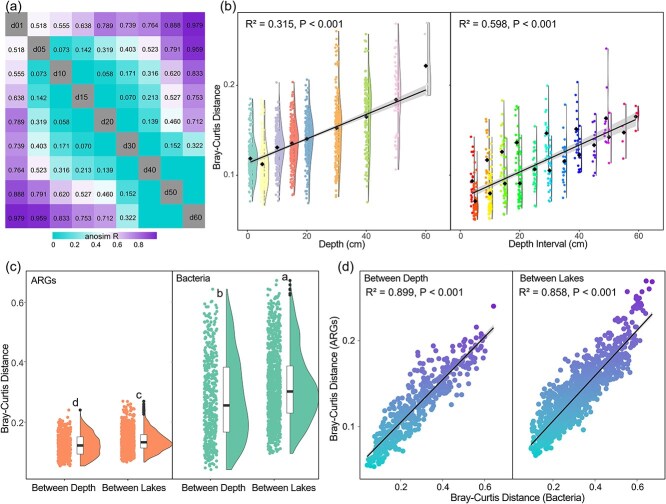
Beta diversity of ARG profiles. (a) ANOSIM *R*-values heatmap showing dissimilarities in ARG profiles between different sediment depths. Only significant (*P* < .05) *R*-values were shown in number. (b) Bray–Curtis distance of ARGs profile plotted against sediment depth and depth intervals (i.e. the vertical distance between paired sampling layers). (c) Comparison of Bray–Curtis distances between depths and lakes for ARGs and bacterial communities. (d) Correlation between Bray–Curtis distances of ARGs and bacterial communities.

The composition of ARGs exhibited pronounced spatial and vertical differentiation across thermokarst lake sediments (Fig. 3). ANOSIM confirmed significant differences in ARG composition among sediment depths (Fig. 3A). Across-lake comparisons revealed that ARG dissimilarity within the same sediment layer increased systematically with depth (Fig. 3B), indicating that deeper sediments host more distinct resistome assemblages. Likewise, dissimilarities among depth intervals within individual lakes increased with depth separation, underscoring the progressive vertical stratification of ARG composition through sedimentary horizons. These patterns reflect niche differentiation driven by sediment stratification, whereby increasing environmental heterogeneity with depth promotes compositional divergence among microbial communities [16, 40]. These results demonstrate that both vertical gradients and geographic isolation jointly structure resistome diversity in thermokarst ecosystems.

Across all study sites, ARG β-diversity was significantly higher among lakes (same depth) than among depths within a lake (Fig. 3C). This suggests that, although vertical zonation structures resistomes locally, between-lake environmental heterogeneity exerts greater influence at regional scales. Distinct physicochemical regimes, such as variations in sediment organic matter, ionic composition, or redox gradients, likely act as environmental filters that shape site-specific resistome signatures [55, 56]. These findings are consistent with canonical ecological theory, whereby geographically isolated habitats develop distinct community compositions through environmental filtering and dispersal limitation [57, 58].

ARG β-diversity was lower than that of bacterial communities (Fig. 3C), although the two were significantly and positively correlated (*P* < .001; Fig. 3D). This pattern suggests that variation in resistome composition is closely linked to shifts in microbial community structure, while remaining more functionally constrained. The relatively lower turnover of ARGs compared to bacterial taxa likely reflects the limited diversity of ARGs and the buffering effect of HGT, which enables ARGs to circulate among phylogenetically diverse hosts [42, 59]. Consequently, microbial dispersal limitation and environmental filtering shape taxonomic differentiation, while HGT promotes functional homogenization of the resistome across taxa and habitats. This reflects a decoupling between microbial phylogenetic turnover and functional gene persistence, a hallmark of functional redundancy and constraint in microbial systems [60]. ARGs with high mobility, often carried on transposons or plasmids, can transcend local microbial boundaries, maintaining a “core resistome” that is shared across ecosystems despite taxonomic divergence [59]. Such dynamics produce a mosaic resistome structure: local environments impose selective pressures that diversify ARG assemblages between lakes, while gene mobility sustains conserved functional modules across vertical and spatial gradients [20].

### Vertical dynamics of mobile genetic elements and their associations with antibiotic resistance genes

**Figure 4:**
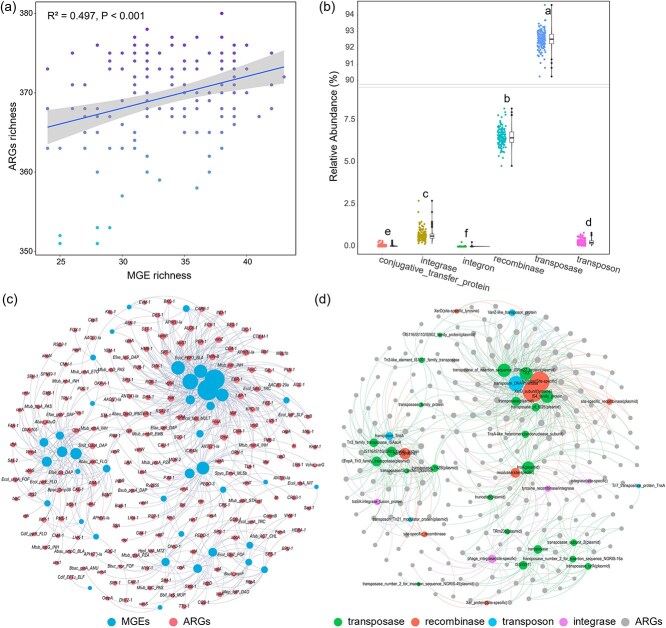
Relationships between MGEs and ARGs in sediment profiles. (a) Positive correlation between MGE richness and ARG richness. (b) Relative abundance of different MGE types. (c and d) Co-occurrence network of MGEs and ARGs.

MGEs act as pivotal vectors of HGT, enabling the dissemination and persistence of ARGs across microbial lineages. In the thermokarst lake sediments, 57 distinct MGEs were identified and classified into six functional categories, encompassing transposases, recombinases, integrases, plasmid-associated elements, prophage components, and insertion sequences. Across all samples, MGE richness strongly correlated with ARG richness (*r* = 0.71, *P* < .001; Fig. 4A), underscoring that HGT is a dominant mechanism maintaining resistome diversity within these sediments [59, 61].

Among the identified MGEs, transposase and recombinase genes overwhelmingly dominated, accounting for 92.5% and 6.5% of total relative abundance, respectively (Fig. 4B). This prevalence of transposases reflects a highly dynamic genomic landscape characterized by recurrent DNA rearrangements and active gene flux. Transposases catalyze the “cut-and-paste” or replicative movement of genetic elements, providing microbial communities with genetic plasticity to adapt to redox and nutrient gradients in stratified sediments [62]. Such mobility not only accelerates the acquisition of resistance genes but also facilitates their long-term maintenance even in environments minimally influenced by anthropogenic antibiotics [51]. The secondary enrichment of recombinases indicates that site-specific recombination contributes to ARG diversification, promoting allelic reshuffling and the potential emergence of novel resistance combinations under environmental stress.

Co-occurrence analysis revealed 214 significant ARG–MGE associations (Spearman *r* > 0.5, FDR < 0.05; Fig. 4C–D), highlighting a dense network of horizontal connectivity. Notably, six highly mobile ARGs, including *APH(6)-Ia, blaS1, FosA, mphA, IreK*, and *vanZ*, each exhibited strong positive correlations with up to nine distinct MGEs, primarily transposase families. This pattern suggests that these ARGs function as “hub genes” within resistome–mobilome networks, capable of rapid propagation across taxa through replicative transposition or composite transposon formation [61, 63]. Core multidrug-resistance genes identified in this study (*abcA, Abau_AbuO, aadT*, and *Van*) were also significantly associated with multiple MGEs, further demonstrating that gene mobility underpins the spatial persistence of broad-spectrum resistance. Beyond simple mobility, these associations imply co-selection between resistance and other adaptive traits. MGEs frequently carry composite cassettes linking ARGs with genes related to carbon metabolism, stress tolerance, or virulence, forming multifunctional genomic islands [64, 65]. This co-mobilization enables simultaneous transfer of metabolic and resistance functions, providing microbes with integrated ecological advantages under fluctuating redox and nutrient regimes. In permafrost-affected sediments, where microorganisms face both energetic limitation and periodic thaw-refreeze stress, such co-selection likely enhances community resilience by linking antibiotic defense with metabolic adaptability [66]. While our network analyses reveal robust co-occurrence patterns, these associations do not imply direct causality. Instead, they likely reflect functional co-selection under shared energetic and redox constraints.

### Functional coupling between antibiotic resistance genes and carbohydrate-active enzymes

**Figure 5:**
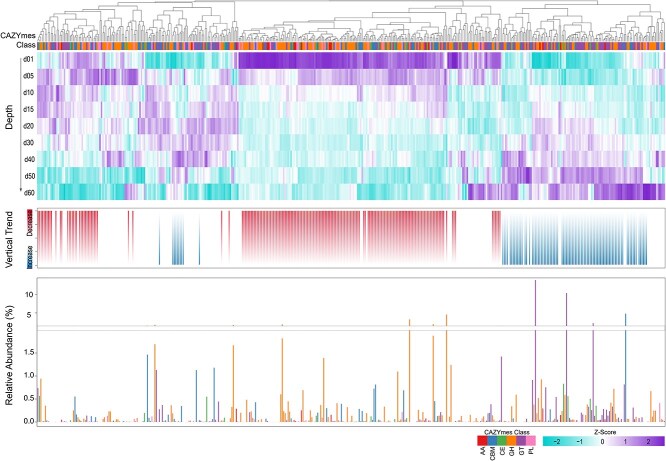
Distribution, relative abundance, and vertical trends of CAZymes across sediment depth profiles.

**Figure 6:**
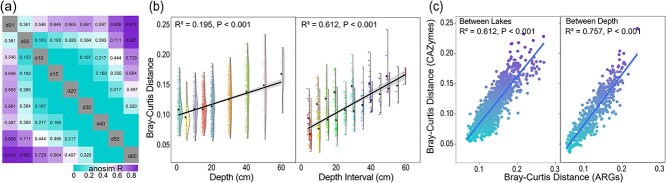
Beta diversity and dissimilarity patterns of CAZymes across sediment depths. (a) ANOSIM *R*-values heatmap showing dissimilarities in CAZyme profiles between different sediment depths. (b) Bray–Curtis distances of CAZyme profiles plotted against sediment depth and depth intervals (i.e. the vertical distance between sampling layers). (c) Bray–Curtis distances plotted against sediment depth (left) and depth intervals (right) for CAZymes.

The metagenomic analyses revealed an exceptionally diverse repertoire of 422 CAZyme families within the thermokarst lake sediments (Fig. 5). These enzymes mediate the degradation of complex polysaccharides such as pectin, glycan, and glucan, forming the metabolic backbone of carbon cycling in these systems. Among all CAZyme groups, glycoside hydrolases (GHs; *n* = 162) and glycosyl transferases (GTs; *n* = 104) dominated the diversity spectrum, followed by carbohydrate-binding modules (CBMs; *n* = 81), polysaccharide lyases (PLs; *n* = 40), carbohydrate esterases (CEs; *n* = 19), and auxiliary activities (AAs; *n* = 16). In terms of relative abundance, GTs (39.2%) and GHs (37.7%) were the most prevalent, while CBMs (16.0%), CEs (4.1%), AAs (1.5%), and PLs (1.5%) were less represented (Fig. S3). The most abundant individual families included GT2 (cellulose synthase), GT4 (sucrose synthase), CBM50, GH13, and GH0, indicating strong potential for both polymer breakdown and cell-wall synthesis. At the vertical scale, 179 CAZymes (78 GHs, 35 CBMs, 24 GTs, 22 PLs, 10 AAs, 10 CEs) decreased with depth, whereas 99 CAZymes (43 GTs, 34 GHs, 13 CBMs, 4 CEs, 3 AAs, 2 PLs) increased (Fig. 5), revealing a metabolic transition from labile-carbon degradation near the surface to recalcitrant-carbon metabolism in deeper strata [22, 67]. These shifts were corroborated by significant compositional differences across both depth and lake gradients (Fig. 6).

The high diversity of GHs and GTs underscores their central role in organic-matter turnover within thermokarst lakes [68]. GHs catalyze polysaccharide depolymerization into soluble sugars, while the abundance of GT2 and GT4, enzymes for cell-wall biosynthesis and modification, indicates active cell-envelope remodeling that supports microbial adaptation under fluctuating redox and nutrient regimes [69]. The prevalence of CBMs, which anchor enzymes to polysaccharide substrates, enhances catalytic efficiency in sediments rich in mineral-bound or structurally protected organic matter [70]. Decreasing CAZyme richness toward depth reflects a decline in microbial exploitation of fresh organic inputs, whereas the enrichment of GTs and CBMs in deeper layers suggests functional specialization toward degradation of refractory compounds such as cellulose and lignin [71, 72]. Such vertical metabolic restructuring is consistent with ecological succession driven by resource limitation, governing the long-term balance between carbon release and sequestration in permafrost-affected lake sediments.

**Figure 7:**
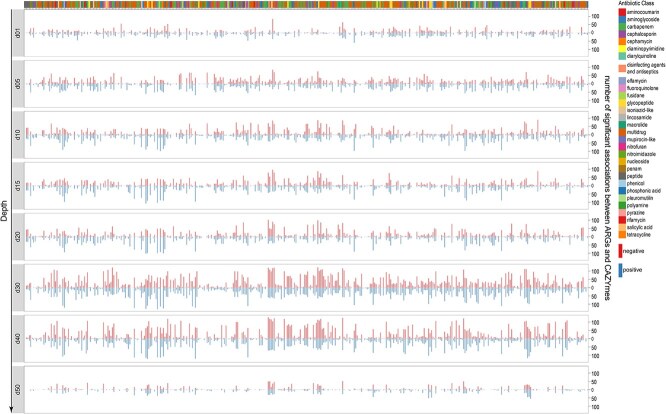
Significant associations between ARGs and CAZymes across sediment depths. Each panel represents a different sediment depth (d01 to d50), showing the number of significant positive and negative associations between ARGs and CAZymes. Antibiotic classes are color-coded to distinguish the types of ARGs.

Our network analyses further suggest that the functional coupling between ARGs and CAZymes in thermokarst lake sediments can be broadly organized into two contrasting but complementary modules (Fig. 7). The first comprises synergistic modules, in which ARGs associated with stress tolerance and cellular homeostasis (e.g. ribosomal protection, target modification, and cell-envelope regulation) co-occur with CAZymes involved in efficient polysaccharide depolymerization. Such associations likely reflect coordinated selection for metabolic efficiency under persistent redox stress and energy limitation, allowing microbial populations to sustain high enzymatic throughput while maintaining resistance to naturally produced antimicrobials and competitive pressures [44, 73]. In this context, antibiotic resistance functions not merely as a defensive trait, but as an enabling component of sustained carbon processing in sedimentary environments. In contrast, trade-off modules are characterized by negative associations between ARGs mediating antibiotic detoxification and CAZymes linked to carbon degradation. Detoxification-based resistance mechanisms, such as enzymatic inactivation or modification of antibiotics, impose substantial energetic and resource costs, potentially constraining investment in carbon-depolymerizing enzymes [74]. These antagonistic relationships point to metabolic allocation conflicts, whereby maintaining resistance through energy-intensive pathways may reduce microbial capacity for organic matter decomposition. These associations likely arise from co-selection under energy-limited and redox-stratified conditions, reflecting canonical ecological principles of environmental filtering and metabolic trade-offs, whereby microbial communities optimize carbon utilization while allocating limited resources to resistance maintenance [16, 60, 64].

Across samples, ARG β-diversity was significantly correlated with CAZyme β-diversity (Fig. 6C), confirming that shifts in resistome composition are closely linked to changes in metabolic capacity. Environmental pressures, such as variable nutrient inputs, antibiotic exposure, and inter-microbial competition, thus jointly modulate both defense and decomposition functions [64]. This co-variation reflects functional co-selection, where ARGs and CAZymes co-occur within mobile genetic modules, allowing microbial populations to synchronize antibiotic resistance with carbon-processing capabilities.

## Conclusion

This study reveals pronounced vertical stratification of ARGs in thermokarst lake sediments on the QTP. Strong associations between ARGs and MGEs indicate that HGT sustains resistance across sedimentary horizons [18, 59]. The significant coupling between ARGs and CAZymes further shows that resistance is metabolically integrated with microbial carbon cycling [60, 64]. These results suggest that ARGs function as adaptive and biogeochemical components rather than isolated resistance traits [5]. With accelerating permafrost thaw, the reactivation and mobilization of ancient resistomes could reshape microbial metabolism and resistance dissemination, linking permafrost degradation to both carbon-cycle feedbacks and emerging environmental risks [22, 31].

## Supplementary Material

ycag107_SI_TL_Vertical_ARGs-20241126

## Data Availability

The data used in this work can be accessed in Zenodo. https://doi.org/10.5281/zenodo.18603126
