# Supplementary material for: Vertical stratification and functional coupling of antibiotic resistance and carbon metabolism in thermokarst lake sediments
Source: ISME Commun. 2026 May 8;6(1):ycag107. doi: 10.1093/ismeco/ycag107 (PMC13184972; doi:10.1093/ismeco/ycag107)
Supplement: ycag107_SI_TL_Vertical_ARGs-20241126 [file ycag107_si_tl_vertical_args-20241126.docx]

## Supplementary Information


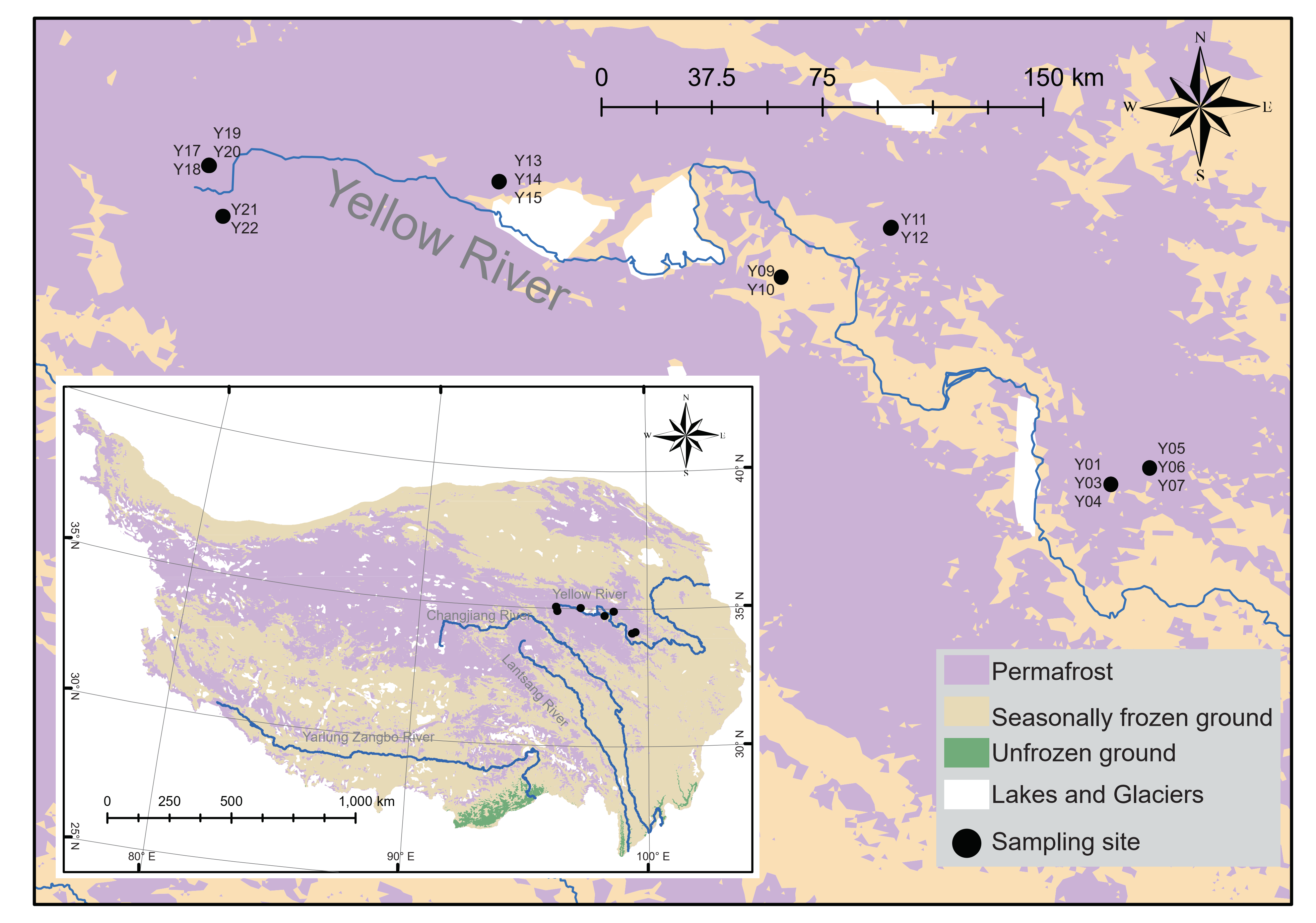


Figure S1 The study lakes are located in the Yellow River Source Area. Sediment cores were collected from 19 lakes.


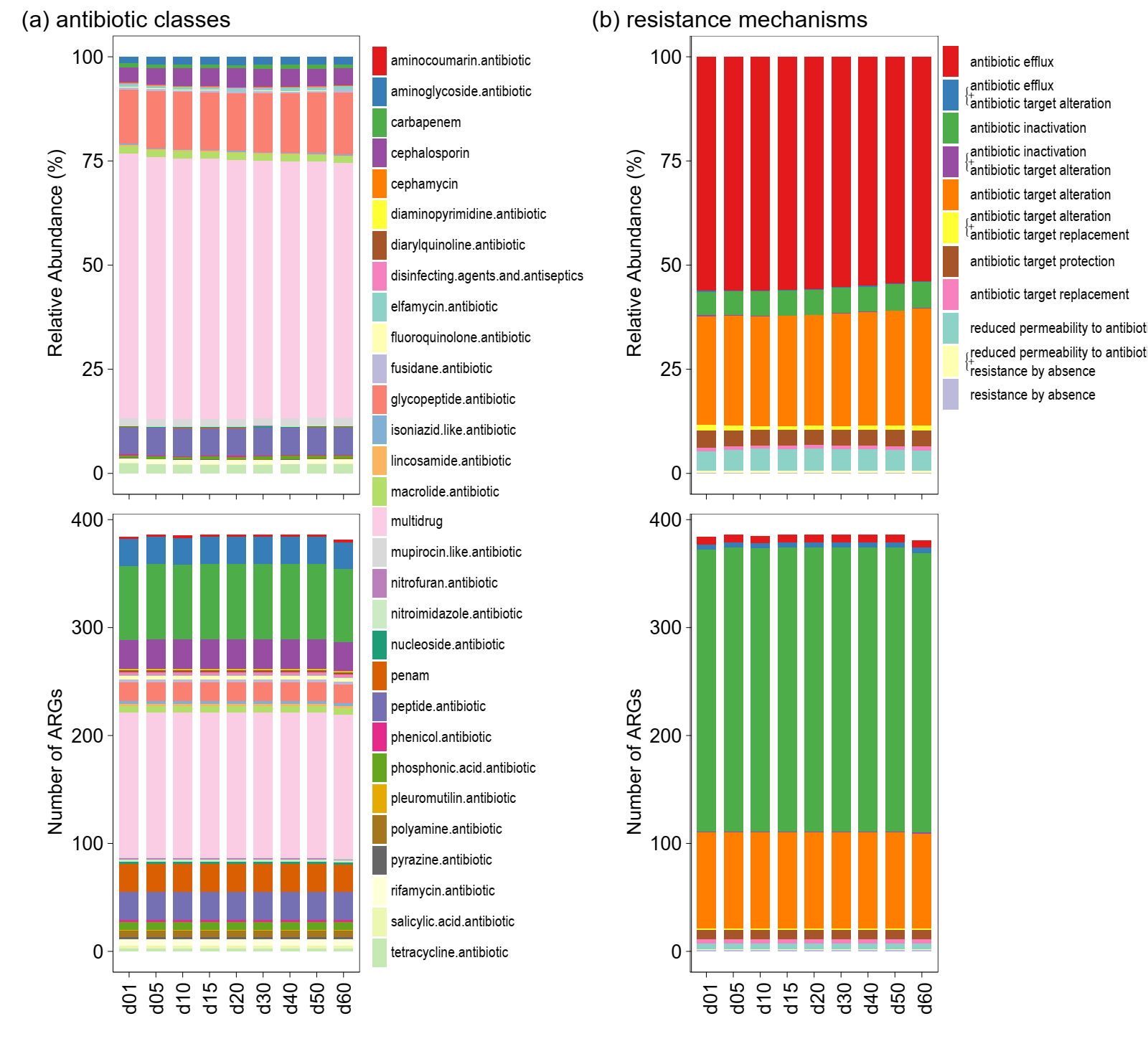


Figure S2 The composition of ARGs at different depth in term of (a) antibiotic classes and (b) resistance mechanism.


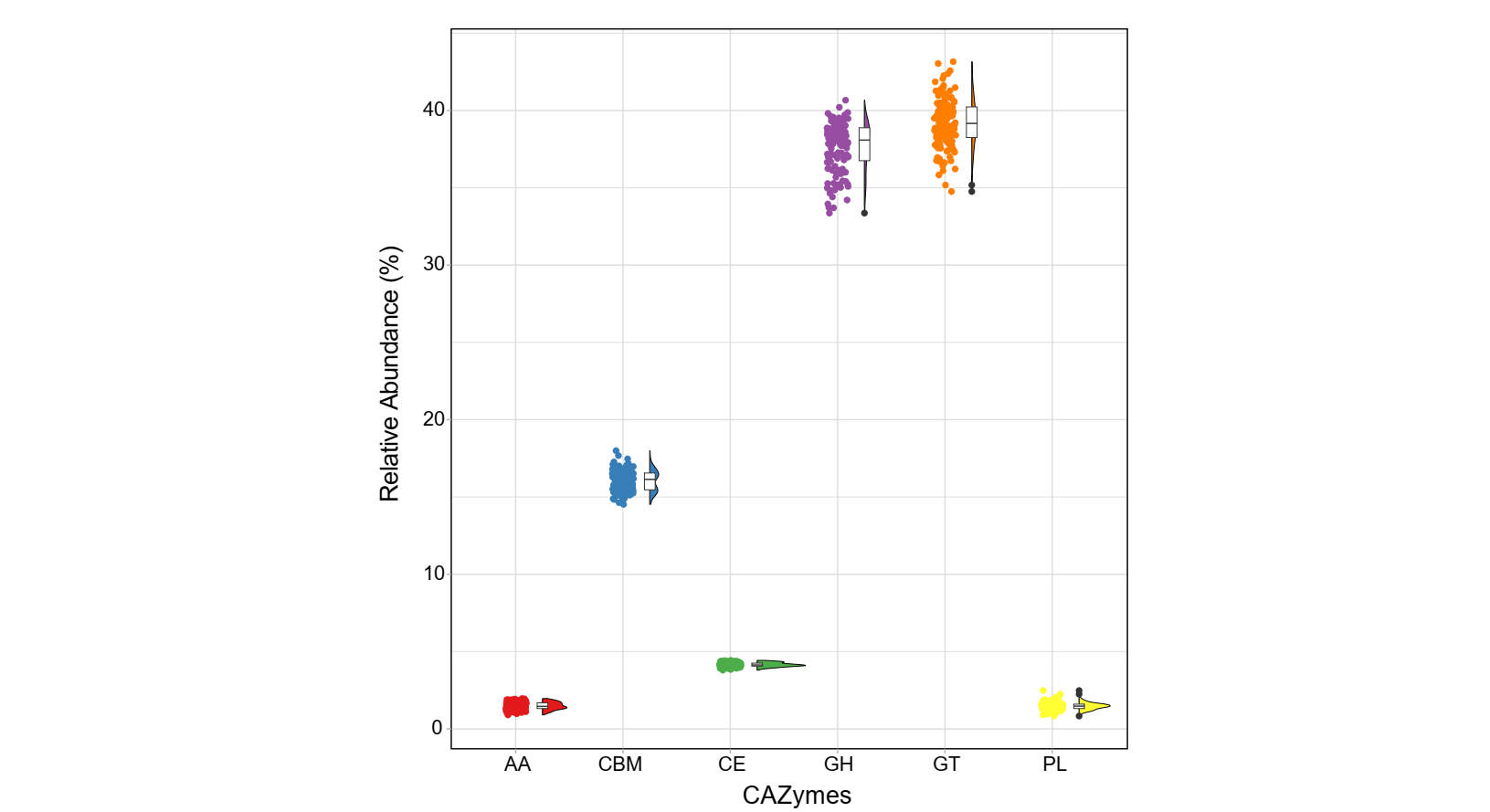


Figure S3 Relative abundance of CAZymes.
